# Medical Opinion and Movement

**Published:** 1908-08-01

**Authors:** 


### Medical Opinion and Movement.

To English visitors and tourists in Italy the present agitation against the right of English doctors to practise in Italian territory on an almost equal footing with the native graduate, is of interest on more than one point. Hitherto any duly qualified practitioner has been allowed to settle in Italy there to follow his profession, and there are at this moment many English and American graduates domiciled in Italy, and in possession of more or less thriving practices. The Italian graduate, however, has not accepted the situation with equanimity, and has pointed out, on more than one occasion, that it is an anomalous condition that the graduate of a country which refuses the privileges of "reciprocity" to Italian medical men, is permitted to compete with the native practitioner, even only so far as the foreign section is concerned. In all other continental countries, the foreign practitioner has to pass a State examination before he is allowed to practise on an equality with the local graduates. In a few countries he is tacitly permitted to practise among his compatriots, travelling or temporarily resident in the country, without having to submit to an examination; and in France, for instance, many English and American diplomates make free use of this privilege. In Italy, however, his activities are not so limited, and the foreign doctor who is qualified to practise in his own country, may, upon complying with certain simple and easy formalities, become entitled to practise anywhere within Italian territory on terms of equality with the native practitioner.

This state of affairs has repeatedly drawn the protests of the faculty in Italy, hitherto without bringing about a change. Now, however, a united appeal has been made, and there is reason to believe that in a short while Italy will very considerably restrict the privileges of foreign doctors practising in Italian towns. The limits of such restrictions are not yet known, but it is only reasonable to suppose that no country which refuses to Italian graduates rights equal to those which Italy grants to outside practitioners will be treated as a "friendly state" so far as this question is concerned. With England Italy is on terms of "medical reciprocity," and any alteration in the present state of things will, therefore, hardly affect English practitioners in Italy, except in so far as it will drive into their hands the patients of other nations who are less reasonable in this matter of reciprocity. No American, German, Russian, Swedish, or Danish practitioner will be able to practise in Italy without taking his M.D. degree of some local university, for none of these countries permits foreign doctors to practise without possessing the "State examination" licence. England, Austria, and France, on the other hand, recognise Italian degrees, the former entirely, and the latter so far as the "foreign colony" in a French health resort is concerned. The present agitation in Italy is one that very seriously affects the interests of practitioners who are subjects of non-reciprocal countries, for there appears to be every expectation that it will be successful, and that Italy will drop into line with other European countries in protecting the interests of her own doctors.

In an article in the *Berl. Klin. Wochenschrift,* Gravitz states that his experience has shown him that the only treatment for leukæmia which is of any benefit consists in the application of *x*-rays. He finds that his results are improved if the patient is kept in bed, is well nourished, and, at the same time, treated with arsenic. The duration of each application and the method used are of great moment. If the case be an old one the morbid tendency in the blood-forming organs is too deeply rooted for normal function to be regained; hence it is important that an examination of the blood be made as soon as any symptoms of leukæmia have shown themselves. A case which commences acutely with septicæmic troubles, and one which shows chronic inflammation of the lymphatic system is of grave prognosis, especially in infants and the aged, for the tissues show a certain incapacity for regaining their normal function. Prognosis is, however, better to-day than formerly, even in the lymphatic type. An analysis of the author's cases shows that out of 44 patients treated, 12 had been recently attacked, and three had relapsed after a year or two of good health. After treatment these all showed signs of improved health. Fourteen chronic cases which had been treated late in the disease showed only one complete cure of two years' standing, but there was marked improvement in all the others except one, which died. Out of 15 cases of the lymphatic variety, one was cured, two died of a recurrence, and the rest were either only slightly improved or displayed an absolute resistance to all treatment.

Dr. Fromaget, in the *Journ. de Med. de Bordeaux*, gives an account of ophthalmia due to exposure to the glare of electric light of great intensity. This affection is accompanied by pain in the eyes, watering, and conjunctival injection. More rarely intense photophobia, blepharospasm, iritis, iridocyclitis, and œdema of the papilla may follow the accident. Usually these symptoms slowly subside and the eye returns to the normal, but prognosis should always be guarded, especially if improvement is much delayed. Prophylactic treatment is not always possible, for though workers who are exposed to the glare of arc lamps and electric flashes can protect their eyes by glasses coloured with uranium salts, tramway-conductors, railway-guards, engine-drivers, etc., cannot use such protection, as their occupation calls upon them to recognise colours. In the case of these latter treatment can only be employed when the affection has declared itself. It consists in soothing the patient by lotions containing stovaine or cocaine, and overcoming congestion by adrenalin. These may be combined in a single lotion. In grave cases where there is optic atrophy treatment is of no avail.

In the continual discussion on the treatment of appendicitis, which has been carried on ever since the disease was first clearly distinguished, the surgical and medical camps have been a little divided on the opinion as to when to operate. While physicians plead for a conservative policy, surgeons often complain that they are called in too late. Both have shown reason on their side, and as a result a middle-line policy has been generally adopted. Never, however, in the course of the discussion has it been suggested that the method of either side was pursued selfishly for personal gain. We hope and believe that any such idea is so contrary to the spirit of the profession, at least in this country, that no member would be found openly and publicly to uphold it. Dr. Kilvin, of New York, however, in a recent issue of the "International Journal of Surgery," contributes an article, which is chiefly distinguished by the base, and we hope baseless charges he makes against his medical colleagues. "One of the chief causes," he says, "for pus formation is the greed of the almighty dollar. The selfish physician holds on to the case until the people have paid out all they have, and not till then informs them that it is a suitable case for operation." He thinks that "it would be wise to educate the public that a pus appendix is the result of an ignorant, careless, or defiant physician." He is, of course, pleading for operation in all cases, but it is surely unnecessary, as well as unjustifiable, to support the extreme surgical view by recourse to such abominable charges against the physician who temporises. We know the dollar is very mighty in the New World, but we hope the medical profession, even there, is not so contaminated by the prevailing taint that treatment of the patient is subservient to its worship. A more suicidal policy to the interests of the profession itself could hardly be conceived.

---

Dr. Milan discusses, in a recent issue of *Le progrès médicale*, the efficacy of mercurial treatment in tabes dorsalis. While admitting the failure of mercury to exercise such a specific action on this disease as on syphilis, he claims to have obtained definite beneficial results in a number of cases. He has collected quite a number of cures reported in medical literature of recent years, but admits that such cases are exceptional and rare. He asserts, however, that about a dozen patients at the present time come regularly to him for a course of mercurial and iodide treatment, and that in no case has the disease progressed since the treatment has been instituted; but in several there has been actual amelioration of certain symptoms. Lightning pains in particular have been favourably influenced by the treatment, although it is admitted that in certain exceptional cases there has been actual exacerbation of the pains during treatment. On the whole, therefore, his experience corroborates that of other authorities that mercury is always worthy of a trial when the disease first comes under observation, although more often than not the results are disappointing. At one time it was suggested that the mercurial treatment of syphilis was actually productive of subsequent tabes. This was, however, definitely refuted by Professor Fournier, who published statistics covering 321 tabetics, all with a history of syphilis. Of these by far the greater proportion had only received very short and inefficient mercurial treatment. These statistics were published in 1891.

---

Dr. Adam H. Wright, professor of obstetrics of Toronto University, has recently discussed in brief outline his methods of treatment of puerperal septicæmia. He naturally lays stress on the importance of early diagnosis, and urges that treatment should be commenced within the first two or three days of the puerperium, upon the slightest symptoms suggesting that things are not quite as they should be. He is thoroughly convinced of the efficacy of calomel and opium, and advocates one grain of calomel every hour for three doses, to be followed in due course with saline cathartics. Opium should be given in sufficient doses to relieve pain. Contrary to the usual teaching, he thinks that to withhold opium from a woman suffering agony from intra-abdominal inflammation because it may "mask symptoms" is "neither scientific, practical, nor humane." With febrile disturbances and a foul lochial discharge on the second or third day an anæsthetic should be administered, the gloved hand introduced into the vagina, with two fingers in the uterus, the debris should be scraped away and washed out with hot saline solution, and the uterus and vagina then packed with iodoform gauze. The packing should be removed within 20 to 30 hours. He deprecates the use of the curette, and expresses the opinion that after the fourth day not even the finger or the douche nozzle should be introduced within the uterine cavity. It is difficult to understand on what grounds such a rule should be laid down, which certainly does not conform to the practice and teaching of the majority of obstetricians on this side. Ordinary sapræmic conditions cannot at once be distinguished from septicæmia. The one condition may develop into the other; moreover, a local sapræmia may develop later in the puerperium, and may readily yield to thorough evacuation and flushing of the uterine cavity. After the first few days, however, Dr. Wright relies on drainage of the uterus by Fowler's position and on rectal injections of saline and collargol.

---

The appointment of Professor Joachimsthal to the chair of orthopædic surgery at the University of Berlin, vacant through the death of Professor Hoffa, was foreshadowed in "The Hospital" some months ago, and the official announcement of Professor Joachimsthal's appointment will be hailed with satisfaction by all who are interested in the progress of orthopædics. At the beginning of the present year there was a well-grounded fear that the authorities favoured the abolition of the special professorship in orthopædic surgery at Berlin. It was said that there was no necessity for such specialisation, and that the subject could be treated equally well by one of the professors in surgery. The vigorous protest of the Orthopædic Congress elicited from the authorities the promise that the chair would be maintained, and that a successor would be appointed before the winter term commenced. In accordance with this undertaking, Professor Lange, of Munich, was "called," but declined the offer, chiefly on account of the fact that Munich contemplates the establishment of the first "State orthopædic institute," while the number of beds at the disposal of the Berlin clinic is limited. The second choice was Professor Joachimsthal, and his acceptance of the call proves that the authorities have been willing to treat the orthopædiac clinic more reasonably and more liberally than in the past Professor Joachimsthal is popular in Berlin both with students and post graduate men, and as an orthopædist he has won a reputation by the excellent work which he has done in various departments of his speciality. Under his direction we feel sure that Berlin will once more become a recognised centre for orthopædic work as it was under his predecessor, Professor Hoffa, and we sincerely congratulate the authorities on the choice they have made.

---

THE twenty-fifth anniversary of the foundation of the New York Post-Graduate Medical School has been celebrated by the publication of a volume of essays and lectures of nearly 500 pages, contributed by many of the best-known physicians and surgeons of that city. Amidst many interesting papers we may note that of Dr. Brodhead, who, having seen a case of rupture of the uterus through the scar of a former cæsarean section, has collected from the literature nineteen other instances of this accident. His deduction is that in repeating cæsarean section labour should be anticipated by a week or ten days, and that if labour begins before operation the latter should be performed as soon as possible. Both suture of the laceration and hysterectomy have proved successful. Dr. Leonard Weber, while admitting tnat there is much to be said for Hunter's theory of oral sepsis as the prime causal factor in pernicious anæmia, attaches importance also to gastro-intestinal toxins, and details four cases which have induced him to take this view.

---

DR. SAMUEL LLOYD writes of the surgical treatment of "unresolved pneumonia," by which he designates empyema. He advocates ether as the best anæsthetic; and an incision in the mid axilla large enough to enable the operator to reach digitally all parts of the lung, including the diaphragmatic surface. A three-inch piece of the sixth rib suffices in children, and a similar length of this and of those above and below in adults. The administration of the anæsthetic is then entirely stopped, and the finger is swept round the collapsed lung so as to separate all pleural adhesions. Coagulated lymph is at the same time loosened, and is swept out by pitcherfuls of normal saline solution. Firm adhesions are cut, and no ill result is said to follow if the manipulations should tear the lung, even into a bronchus. As the patient comes round gradually from the ether, digital stimulation of the pulmonary pleura sets up a hacking cough, the result of which is to dilate the collapsed lung. Lymph coagula are to be curetted away with a spoon if necessary; and all blood clot is washed out of the pleura, a process which Dr. Lloyd has not found attended with bad results. A very short spool drainage tube is used, of similar pattern to that which Mr. Bilton Pollard has been using for some years in this country. Two other very exhaustive contributions are those by Drs. Jacobson and Brothers, respectively on primary ovarian pregnancy and atresia vaginæ.

---

DR. GROSSE is very emphatic on the "hydriatic" management of burns, first advocated ninety years ago by Dzondi, of Halle, and later by Winternitz. Briefly Dr. Grosse advises a continuous warm bath for variable periods as the best initial treatment of a burn. If a limb is affected, it is immersed in a suitable vessel; if the trunk, the whole body is put into a bath-tub. The clothing, unless loose, is not removed, but the affected area, just as it is, is enveloped in water at a temperature of 80° to 93° F. The temperature is governed partly by the patient's own sensations, partly by the extent of the burn; large burns require higher temperatures than small ones. The pain is thus relieved, and when this is brought about the bath may be heated to 99° if thought fit, or a water dressing may be applied. Blisters are left intact. The first dressing consists of two or three layers of wet muslin, linen, or gauze, which are left undisturbed for 24 to 48 hours. Over them cool compresses are applied, and renewed at once as soon as pain returns; this is at first sometimes once a minute, but the interval soon lengthens; no ice is used. Later on very dilute antiseptics are used instead of water for the dressings, such as boric acid, one-third of a concentrated solution; protargol, ¼ per cent., and so on. The temperature of these solutions should be between 75° and 85°, and the patient's sensations are a trustworthy guide. The gauze should be cut to fit the burnt area, and healthy skin covered with protective. The dressing is changed every one to eight hours, according to the amount of discharge. The heroic treatment by scrubbing with ether and strong antiseptics under anæsthesia is severely condemned.

---

IT sometimes happens that children who are being artificially fed show signs that their food is not agreeing with them, although every precaution is being taken both as to the quantity and quality of the milk given. Giliberti attributes these troubles to the introduction into the organism of a heterogeneous albumin. Breast-fed children are immune to these troubles, because the albumin they receive is homogeneous. The author is of opinion that it is possible to prevent these nutritional disturbances while continuing artificial feeding, by injecting subcutaneously serum derived from an animal of the same species as the one which is supplying the milk. Thus, in the case of a child fed on cow's milk, the author has given hypodermic injections of cow's serum, the result being that the digestive troubles have ceased, and the child has been able to continue on artificial feeding.

